# Does Duloxetine Improve Cognitive Function Independently of Its Antidepressant Effect in Patients with Major Depressive Disorder and Subjective Reports of Cognitive Dysfunction?

**DOI:** 10.1155/2014/627863

**Published:** 2014-01-19

**Authors:** Tracy L. Greer, Prabha Sunderajan, Bruce D. Grannemann, Benji T. Kurian, Madhukar H. Trivedi

**Affiliations:** Department of Psychiatry, The University of Texas Southwestern Medical Center, Dallas, TX 75390-9119, USA

## Abstract

*Introduction*. Cognitive deficits are commonly reported by patients with major depressive disorder (MDD). Duloxetine, a dual serotonin/noradrenaline reuptake inhibitor, may improve cognitive deficits in MDD. It is unclear if cognitive improvements occur independently of antidepressant effects with standard antidepressant medications. *Methods*. Thirty participants with MDD who endorsed cognitive deficits at screening received 12-week duloxetine treatment. Twenty-one participants completed treatment and baseline and posttreatment cognitive testing. The Cambridge Neuropsychological Test Automated Battery was used to assess the following cognitive domains: attention, visual memory, executive function/set shifting and working memory, executive function/spatial planning, decision making and response control, and verbal learning and memory. *Results*. Completers showed significant cognitive improvements across several domains on tasks assessing psychomotor function and mental processing speed, with additional improvements in visual and verbal learning and memory, and affective decision making and response control. Overall significance tests for executive function tasks were also significant, although individual tasks were not, perhaps due to the small sample size. Most notably, cognitive improvements were observed independently of symptom reduction on all domains except verbal learning and memory. *Conclusions*. Patients reporting baseline cognitive deficits achieved cognitive improvements with duloxetine treatment, most of which were independent of symptomatic improvement. This trial is registered with NCT00933439.

## 1. Introduction

Cognitive deficits are commonly reported by patients with major depressive disorder (MDD) [1, 2] and yet they remain poorly understood. The impairments most commonly associated with MDD are in the domains of executive function, selective attention, and verbal learning [1, 3]. On the other hand, generalized psychomotor slowing [4, 5] can also impact performance across several cognitive processes and domains. Impaired cognition is likely associated with difficulties in everyday tasks contributing to the high degree of psychosocial and functional impairments [6–8], reduced productivity [9], and disability associated with MDD [10, 11]. For example, difficulties with planning and organization can significantly impair daily activities such as one's ability to take care of family-related matters (e.g., childcare or managing finances) or one's ability to perform efficiently on the job [12]. In fact, a recent study examining performance-based assessment of functional skills (e.g., paying a bill or rescheduling an appointment) in Chinese participants with severe mental illness found that functional skills were significantly impaired in depressed individuals compared to healthy control participants [13]. Because cognitive impairments occurring with MDD have been associated with persistent functional impairment and disability, they warrant focused attention during the treatment of depression.

Cognitive benefits have been noted with the most commonly utilized antidepressants, although the results of these studies are inconsistent. Treatment with selective serotonin reuptake inhibitors (SSRIs) has resulted in improved attention, memory, and learning [14], and similar results have been obtained when comparing cognitive performance following treatment with fluoxetine or the selective noradrenergic reuptake inhibitor, reboxetine [15]. However, these studies have been primarily focused on older depressed individuals and/or late onset depression. One study has previously demonstrated improvements in cognitive function with duloxetine treatment, with effects limited primarily to improved verbal learning, but this study was also focused on elderly patients with MDD [16].

In studies conducted with younger depressed subjects, SSRI treatment again yielded cognitive improvements in declarative memory, psychomotor speed, and attention, and some evidence suggests that benefits obtained with SSRI are superior to those seen with tricyclics [17, 18]. In contrast, there are many reports of cognitive impairments that remain following antidepressant treatment that successfully reduces depressive symptomatology [19–21], even when remission of depressive symptoms is obtained [22–24]. As with many other residual symptoms of depression, such as insomnia, fatigue, or somatic symptoms, residual cognitive impairment may contribute to increased risk of relapse, impaired quality of life, and poorer overall prognosis [6, 25, 26].

The adverse consequences of cognitive impairment underscore the need to identify treatments that directly improve cognitive function specifically in adult depressed patients who present with cognitive impairment [25, 27]. The availability of the newer dual serotonin and noradrenaline reuptake inhibitors (SNRIs) may be of particular benefit to individuals with cognitive dysfunction as a part of their depression, given the targeted noradrenergic mechanism of action of these agents. Duloxetine is a well-tolerated, efficacious [28–30] SNRI antidepressant that has been shown to increase extracellular 5-HT, norepinephrine, and dopamine in rat frontal cortex [31] and has been associated with serotonergic and noradrenergic reuptake in humans [32]. Although much previous work on cognitive functioning has stressed the importance of intact dopaminergic levels, particularly with respect to functions mediated by prefrontal cortex, such as working memory, it is now being recognized that norepinephrine plays a similarly important role [33] and that both neuromodulators have distinct roles to facilitate information processing in the prefrontal cortex [33, 34].

Recently, Herrera-Guzmán et al. [35, 36] compared cognitive function following treatment with either duloxetine or escitalopram and found that both improved declarative memory, working memory, set shifting, spatial planning, mental processing speed, and motor performance. Interestingly, duloxetine treatment resulted in greater improvements in declarative and working memory than those achieved with escitalopram [35], suggesting a potential superior benefit of SNRI compared to SSRI for cognitive impairments in MDD. In addition, episodic memory and processing speed improvements were reported to be at least partially independent of improvements in depressive symptom severity [35]. This is an important issue, because there is evidence suggesting that cognitive impairments may be independent of symptom severity [37], but there is also evidence suggesting that changes in cognitive function are associated with symptomatic improvement [15, 38, 39]. However, these studies were not explicitly designed to test for this relationship, and therefore are limited in their ability to explain the relationship between symptom severity and cognitive improvements. Furthermore, the Herrera-Guzmán [35, 36] studies excluded individuals with an incomplete response to treatment (defined as less than 50% reduction of Hamilton Rating Scale for Depression, 17-item [HRSD_17_] score at Week 4), suggesting that the potential effects of duloxetine on cognitive function and the association with those changes to symptom severity should be investigated across a full range of treatment responses as opposed to a narrowly defined subgroup.

The primary objectives of this study were (1) to assess the effect of duloxetine treatment on cognitive function, using the Cambridge Neuropsychological Test Automated Battery (CANTAB; Cambridge Cognition 2004) [40] in young- to middle-aged adults with MDD, and (2) to examine the relationship between changes in depressive symptom severity and changes in cognitive function. A unique feature of this study design was the entrance criterion requiring participants to subjectively report significant cognitive impairment. In addition, the use of a computerized testing battery allowed for the examination of processing and response times, in addition to performance measures (e.g., percent correct, percent errors, etc.). The cognitive domains selected for assessment in this study are those that are most consistently reported as impaired in depression (attention, visual memory, executive function, decision making and response control, and verbal learning and memory). Thus, we examined a wide range of cognitive abilities that we hypothesized would improve with duloxetine treatment. We also hypothesized that cognitive improvements would be related to improvements in symptom severity. Limited information exists regarding whether effects on cognitive deficits can be seen independently of the antidepressant effect. We believe direct investigation of the effect of duloxetine on cognitive function in those who directly report cognitive deficits is important to the field in that it may help guide clinicians toward a treatment option that will improve depression in general, while also specifically targeting cognitive deficits associated with depression.

## 2. Methods

### 2.1. Participant Selection

Participants were recruited from the community and from physician referrals. The protocol and related documents (including informed consent) were approved by the University of Texas Southwestern Medical Center at Dallas Institutional Review Board. Prior to their participation in the study, participants were given a full explanation of the study procedures and possible side effects of the study medication, and they had the opportunity to have any questions answered about the study or study participation. All participants provided written informed consent.

Our target population was young- to middle-aged persons with MDD who reported difficulties with concentration and/or cognition as a part of their depressive syndrome, as measured by a score of 2 or greater on the Inventory for Depressive Symptomatology (IDS-C_30_) [41] item addressing this symptom (#15: Concentration and Decision Making, which is scored based on the following anchor points: 0—“no change in usual capacity to concentrate and decide”; 1—“occasionally feels indecisive or notes that attention often wanders”; 2—“most of the time struggles to focus attention or make decisions”; and 3—“cannot concentrate well enough to read or cannot make even minor decisions.”). Specific additional inclusion criteria were as follows: (1) ability and willingness to provide written informed consent; (2) primary diagnosis of nonpsychotic MDD; (3) age 18–45; (4) Screening and baseline Hamilton Rating Scale for Depression 17-item (HRSD_17_) [42] score greater than or equal to 16 or Clinical Global Impression Scale Severity (CGI-S) [43] score of at least 4; and (5) abstinence of alcohol and hypnotics for 12 hr prior to cognitive testing.

Exclusion criteria were as follows: (1) presence of significant comorbid medical condition based on laboratory test, physician information, or evidence at examination; (2) participant report or evidence (based on physical examination or laboratory tests) of existing liver disease; (3) presence of other psychiatric disorders that were not secondary to depression or that constituted high risk; (4) concomitant pharmacological or psychotherapeutic treatment (those expected to affect depressive symptoms or cognition); (5) failure to respond to 2 adequate courses of antidepressant in the current episode (as measured by the Antidepressant Treatment History Form [44]); (6) hospitalization for mental illness within the past year; and (7) currently pregnant, planning to become pregnant in the next year, or breastfeeding.

### 2.2. Screening

The Structured Clinical Interview for DSM-IV Axis I Disorders, Clinician-Rated version (SCID-CV) [45], was administered to all potential participants to diagnose MDD and rule out excluded comorbid psychiatric disorders. The Antidepressant Treatment History Form [44] was used to assess previous treatment courses for the current episode. Participants received hematology, blood chemistry, thyroid function tests, liver function tests, urinalysis, and urine drug screen to rule out excluded medical conditions. A urine and/or serum pregnancy test was performed as clinically indicated, for all women of child-bearing potential. Participants met with a psychiatrist for a physical evaluation and confirmation of diagnoses.

### 2.3. Assessments

#### 2.3.1. Cognitive Function

The National Adult Reading Test—Revised (NART-R) [46] was given at baseline to assess premorbid intelligence. Clinician-rated and self-reported cognitive function was assessed via the Inventory for Depressive Symptomatology item “Concentration and Decision Making”. CANTAB was used to assess pre- and post-treatment cognitive function and was administered by raters who were blinded to symptom severity assessments. CANTAB is a comprehensive neuropsychological testing battery that has been used to assess cognitive function in a wide variety of brain disorders, including mood disorders. Tasks were selected for use in this study based on their previous exhibition of differences in depressed patients as compared to healthy controls. The tasks selected represented each of the following domains, which were used to group task performance during analyses: Attention, Visual Memory, Executive Function/Set-Shifting and Working Memory, Executive Function/Spatial Planning, Decision Making and Response Control, and Verbal Learning and memory. In addition, these tasks measure specific domains (attention, perception, working memory, declarative memory, and effortful control) that are consistent with those included in the National Institute of Mental Health Research Domain Criteria (NIMH RDoC) initiative [47], which aims to reassess pathophysiology of chronic mental illnesses through a dimensional approach [48]. An overall description of each task assessed in this study is provided below.

*(1) Attention Domain Tasks*. Motor screening (MOT) screens for visual, movement and comprehension difficulties; big circle/little circle (BLC) is a simple attention measure that tests comprehension, learning, and reversal of a rule; reaction time (RTI) measures speed of response to both predictable and unpredictable visual stimuli.

*(2) Visual Memory Domain Tasks*. Delayed matching to sample (DMS) is an object recognition task using complex visual patterns in which the choice is presented either simultaneously with the sample or after a brief delay; paired associates learning (PAL) is a delayed response visual memory and learning task; pattern recognition memory (PRM) assesses visual spatial recognition memory.

*(3) Executive Function/Set-Shifting and Working Memory Domain Tasks*. Intradimensional/extradimensional shift (IED) examines set-shifting and flexibility of attention by testing both simple and more complex rule acquisition and reversal; spatial working memory (SWM) is an executive function task assessing retention and manipulation of items in working memory, with the ability for assessment of perseverative (redundant) errors.

*(4) Executive Function/Spatial Planning Domain Task*. Stockings of Cambridge (SOC) is an executive function task based on the Tower of London test that assesses spatial planning.

*(5) Decision Making and Response Control Domain Task*. Affective go/no-go (AGN) assesses information processing biases and inhibitory control for positive and negative stimuli.

*(6) Verbal Learning and Memory Domain Task*. Verbal recognition memory (VRM) is a measure of immediate and delayed verbal recall and recognition.

Each task can generate multiple outcome measures (e.g., percent correct and response latency), as described in Table 1.

#### 2.3.2. Symptom Severity

The Hamilton Rating Scale for Depression, 17 item, (HRSD_17_) [42], and the 30-item Inventory for Depressive Symptomatology–Clinician-Rated (IDS-C_30_) [41] were administered by trained evaluators to assess severity of depressive symptoms. The 30-item Inventory for Depressive Symptomatology–Self-Report (IDS-SR_30_) [41] was used to assess self-reported depressive symptoms. The IDS is a 30-item, depression-specific symptom severity rating scale, designed to measure the specific signs and symptoms of depression, including melancholic and atypical features. Symptom severity measures were collected at each study visit by evaluators who were blind to the results and status of cognitive testing.

### 2.4. Medication Management

Participants began open-label 12-week treatment with duloxetine at a starting dose of 30 mg per day for the first four days of treatment and then increased to 60 mg per day. Dosage changes were allowed at Weeks 2, 3, 6, and 9 to a maximum of 120 mg per day depending on the side effect profile, tolerability, and symptomatology. Dosage remained constant during Weeks 9–12 of the study, unless side effects or safety warranted a change. Decisions regarding changes in dose were guided by the principles of measurement-based care (MBC) [49] and aided by administration of the 16-item Quick Inventory for Depressive Symptomatology–Clinician-Rated (QIDS-C_16_). Scores ≤5 resulted in continuation of the current dose, scores between 6 and 8 allowed the clinician to choose whether or not to increase or maintain the current dose, and scores ≥9 resulted in a dose increase. These MBC principles were used successfully in the Sequenced Treatment Alternatives to Relieve Depression (STAR*D) study [49, 50].

Participants met the study psychiatrist at all study visits to assess suicidality, side effects, adverse events, alcohol consumption, and improvement. In addition to the QIDS, the psychiatrist administered the Clinical Global Impression Scale (CGI) [43] to assess the overall impression of the participants' symptom severity and overall change from baseline. The Frequency, Intensity, and Burden of Side Effects Rating (FIBSER) [51] and Patient Rated Inventory of Side Effects (PRISE) scales were used to assess the frequency, intensity, and degree of functional impairment associated with side effects of duloxetine and the specific types of adverse effects (e.g., sleep and sexual function). Liver function tests were repeated during the course of the study at the study visit following a dose increase to 90 or 120 mg, or if symptoms of liver toxicity were present or suspected at any time during the study.

### 2.5. Statistical Analyses

Prior to conducting analyses all cognitive testing outcome measures (see Table 1) were examined for normality. Seven measures were removed from subsequent multivariate analyses because of lack of variation in the task performance (e.g., ceiling or floor effects): BLC mean percent correct, DMS percent correct (simultaneous), and IED stages completed and four VRM measures: immediate free recall total novel words, immediate free recall total perseverations, immediate recognition total false positives, and delayed recognition total false positives. Four additional measures were removed from subsequent analyses—the SOC mean subsequent thinking time for 2-, 3-, 4-, and 5-move problems—because many people took longer to physically move the balls than they did to think about the moves, resulting in the absence of a calculation for this measure. This was most common for 2-move problems and decreased with each increasing number of moves.

The remaining measures from the CANTAB battery that met analysis criteria were grouped into sets based on the cognitive domains they represented and were analyzed via separate multivariate analyses (MANCOVA and/or MANOVA, as described below) to account for multiple comparisons. In addition, this also gives the advantage of allowing examination of a collection of highly related variables together, providing additional sensitivity to changes within that domain, which can be followed by separate analyses to determine if the effect is general or associated with a particular test or tests. Therefore, if a significant overall effect was found, secondary analyses (i.e., *t*-tests) were conducted to determine significance for individual measures. In order to determine the extent to which changes in cognition are associated with changes in symptom severity, a hierarchical analysis approach was utilized [52]. The first step was to use a MANCOVA which included depression symptom severity (based on HRSD_17 _score) as a covariate. If no changes were found, the second step involved conducting the analyses with the covariate removed. Note that age, education, and estimated intelligence were not needed because participants served as their own control. Normed scores were provided when calculated by CANTAB.

## 3. Results

Sixty-four individuals signed informed consent and were screened for participation in the study. Thirty-four individuals were excluded during the screening process for the following reasons: presence of significant comorbid medical condition or abnormal laboratory values (*n* = 12), presence of other psychiatric disorder or severity of symptoms (*n* = 9), declining further participation (*n* = 2), failing to follow up (*n* = 8), outside age criterion (*n* = 1), and prohibited concomitant medication (*n* = 2).

Thirty participants were found to be eligible for the study, received a baseline evaluation, and began study medication. Nine participants withdrew prior to study completion. Six had intolerable side effects (two had excessive sedation, one had an allergic reaction, one had insomnia, one had fatigue and insomnia, and one had fatigue, insomnia, and decreased appetite). One serious adverse event occurred for a participant who had acute pancreatitis secondary to gallstone obstruction. This participant had elevated liver enzymes that resolved after medical management of the event. The participant was discontinued from study participation and referred to follow-up care for depression. Two participants withdrew consent and were lost to followup.

Twenty-one participants completed all 12 weeks of study participation. The mean medication dose at Week 12 was 90 mg/d (±28.5). One participant continued throughout the study at 30 mg and was not raised to 60 mg at the discretion of the physician; however, data was included in the analysis based on the intent-to-treat principle. Baseline demographic and clinical characteristics of these individuals are presented in Table 2.

### 3.1. CANTAB

**Figure 1:**
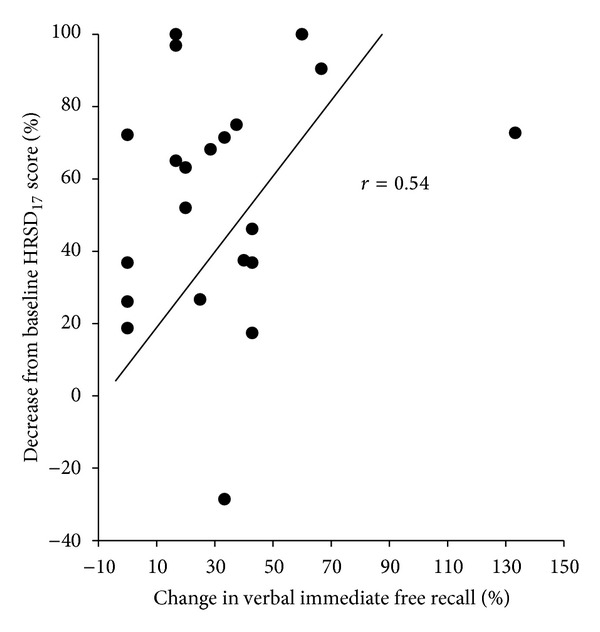
Correlation between percent decrease from baseline on the Hamilton Rating Scale for Depression, 17 items (HRSD_17_), and the immediate free recall total correct verbal learning and memory task. The greater the percent decrease from baseline on HRSD_17_, the more words recalled on the verbal learning task.

The MANCOVA for verbal learning and memory revealed a significant relationship between change in symptom severity and change in verbal learning and memory (time × severity, *F*(1,19) = 12.1, *P* < .003; time × severity × verbal learning and memory, *F*(2,18) = 0.60, *P* < .56). To understand the nature of this effect, we conducted additional MANOVAs to examine the relationship between change in HRSD_17_ and each of the verbal learning and memory tasks. The immediate free recall task was significantly associated with change in HRSD_17_ (time × severity, *F*(1,19) = 11.5, *P* < .004), with a correlation between percent change in HRSD_17_ and percent change in immediate free recall of *r* = .54 (see Figure 1). The two verbal recognition tasks (both immediate and delayed) were not significantly associated with change in symptom severity.

The results of the MANCOVAs using percent change in HRSD_17_ as the measure of symptom severity change produced no significant effects in the remaining cognitive domains: Attention (Time × Severity, *F*(1,19) = .17, *P* < .69; Time × Severity × Attention, *F*(2,18) = .34, *P* < .72), Visual Memory (Time × Severity, *F*(1,19) = .22, *P* < .65; Time × Severity × Visual Memory, *F*(7,13) = .55, *P* < .79), Executive Function/Spatial Planning (Time × Severity, *F*(1,19) = 1.27, *P* < .28; Time × Severity × Executive Function/Spatial Planning, *F*(6,15) = .58, *P* < .72), Decision Making and Response Control (Time × Severity, *F*(1,19) = .13, *P* < .73; Time × Severity × and Decision Making and Response Control, *F*(6,15) = 1.43, *P* < .27). Therefore the symptom severity covariate was dropped from the analysis and only the MANOVAs are reported below, indicating that the changes in cognitive function are independent of change in symptom severity.

**Figure 2:**
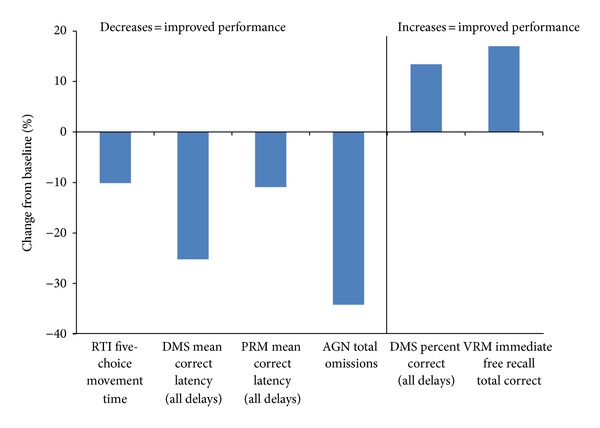
Group percent change from baseline on tasks that significantly improved with duloxetine treatment is presented to give an indication of the magnitude of changes observed. RTI: reaction time; DMS: delayed matching to sample; PRM: pattern recognition memory; AGN: affective go/no-go; VRM: verbal recognition memory.

Significant domain-by-time interactions were obtained for all of the cognitive domains assessed: Attention (*F*(2,19) = 68.79, *P* < .0001), Visual Memory (*F*(7,14) = 76.64, *P* < .0001), Executive Function/Set-Shifting and Working Memory (*F*(5,16) = 80.58, *P* < .0001), Executive Function/Spatial Planning (*F*(4,17) = 15.95, *P* < .0001), and Decision Making and Response Control (*F*(2,19) = 802.04, *P* < .0001). Post hoc analyses revealed significant improvements on specific tasks within 3 of the cognitive domains—(*t* value, and significance level reported in parentheses; see Table 3 for additional details): Attention domain—reaction time five-choice movement time (*t* = −3.48, *P* = .002); Visual Memory domain-delayed matching to sample percent correct (all delays) (*t* = 5.00, *P* < .0001), delayed matching to sample mean correct latency (all delays) (*t* = −3.20, *P* = .005), and pattern recognition memory mean correct latency (*t* = 2.27, *P* = .03); and Decision Making and Response Control domain-affective go/no-go total omissions (*t* = −2.71, *P* = .01). In order to illustrate the magnitude of changes observed, group percent change from baseline on tasks that significantly improved with duloxetine treatment are depicted in Figure 2. Affective go/no-go total commissions (*t* = −1.96, *P* = 0.06) and immediate free recall total novel words (*t* = −2.03, *P* = 0.06) and immediate recognition total correct (*t* = 2.02, *P* = 0.06) measures approached significance. The overall effects in the Executive Memory (Set-shifting and Working Memory and Spatial Planning) domains indicate that there were combined effects when all measures are examined collectively, but the lack of significance for any individual tests may indicate there was insufficient power to detect significant differences for any given individual task alone.

### 3.2. Subjective Cognitive Function

Clinician-rated evaluation of cognitive function (based on the Inventory for Depressive Symptomatology item 15—Concentration and Decision Making) also improved following 12 weeks of duloxetine treatment. The mean item 15 score decreased from 2.14 (±0.36) at baseline to 0.62 (±0.74) after treatment. Similarly, the IDS Self-Report item (#15) decreased from a mean of 2.05 (±0.76) to 0.52 (±0.68), also indicating subjective cognitive improvement.

### 3.3. Symptom Severity

Changes in HRSD_17_ over the 12 weeks of treatment showed that participants' depressive symptom severity improved significantly, with a mean score of 19.4 (4.4) at baseline, dropping to a mean score of 8.3 (5.7) at Week 12, corresponding to an average decrease of 54.5% (*P* < .0001). The IDS-C_30_ and IDS-SR_30_ scores showed similar decreases from baseline mean scores of 36.8 (8.7) and 37.9 (12.1), respectively, to Week 12 scores of 14.7 (10.0) and 13.7 (11.4). Twelve of the 21 completers (57.1%) were defined as responders (i.e., a 50% or more decrease in the HRSD_17_), and those same individuals were remitters, defined by a score of 7 or less on the HRSD_17._

### 3.4. Adverse Effects

Participants completed the PRISE at each study visit, which assess participants' reports of adverse effects in a variety of categories, regardless of whether or not the effects are attributed to medication. Adverse effects most commonly reported by participants who completed the study (*n* = 21; reported as percent occurrence across all observed visits (*n* = 145)) included difficulty sleeping (55.9%), headache (44.1%), poor concentration (43.5%), fatigue (43.5%), anxiety (40.0%), dry mouth (33.8%), decreased energy (33.1%), and loss of sexual desire (30.3%). Side effects occurring between 20.0–29.9% of visits included restlessness (29.7%), constipation (21,4%), increased perspiration (20.7%), and trouble achieving orgasm (20.0), and those occurring between 10.0–19.9% included frequent urination (19.3%), general malaise (18.6%), ringing in ears (17.2%), dizziness (17.2%), blurred vision (16.6%), dizziness on standing (15.1%), itching (15.1%), sleeping too much (14.5%), nausea/vomiting (13.8%), dry skin (11.7%), and diarrhea (11.7%). Side effects occurring less than 10% included tremors (9.7%), menstrual irregularity (7.6%), heart palpitation (6.2%), rash (6.2%), poor coordination, (5.5%), chest pain (4.8%), painful urination (2.1%), and difficulty urinating (1.4%). It should be noted that some effects listed on the PRISE overlap with depressive symptomatology. On the FIBSER, the mean score for frequency of side effects attributed to duloxetine in Week 1 was 2.5 ± 2.0, which corresponds to a rate of 37% of the time. Mean score for severity was 2.7 ± 1.7, which is less than moderate, and interference with function was 2.0 ± 1.6, corresponding to “mild.” At Week 12, side effects were less frequent (mean score = 1.5 ± 2.0, corresponding to 17.5% of the time), less severe (mean score = 1.1 ± 1.7, corresponding to slightly over “trivial”) and interfered less with function (mean score = 0.5 ± 1.0, corresponding to less than “minimal”).

## 4. Conclusions

In this study, young-to middle-aged adults with MDD endorsing problems with concentration and/or decision making at presentation showed significant improvements in cognitive function after 12 weeks of treatment with duloxetine. The aspects of cognitive function that improved predominantly involved psychomotor speed (both movement and thinking). Specific improvements were also noted on tasks assessing visual memory, decision making/response control for emotionally laden information, and verbal recognition memory. As expected, there was also a significant reduction in depressive symptoms with treatment. With the exception of the verbal immediate free recall task, cognitive improvements were independent of improvements in depressive symptom severity. These data suggest that persons who subjectively report cognitive impairment as a part of their depressive syndrome may realize cognitive improvements with duloxetine treatment. Specifically, psychomotor and mental processing speed may be improved with duloxetine treatment, spanning across several types of cognitive domains, and these improvements are not solely related to depressive symptom reduction.

Tasks in this study that significantly improved following duloxetine treatment yielded percent improvements ranging from 10% to 34%. It should be noted that while percent improvement is presented to help provide some equivalence of performance changes across tasks, a small percent improvement may be more important for one type of task than a larger improvement in another task. For example, small decreases in performance time that may be realized with treatment can be clinically meaningful, as cumulative decreases in the time needed to perform tasks can result in improved efficiency for depressed persons. Improved efficiency among depressed persons is important given the decreased productivity that is associated with MDD. The decreases in performance time that were observed following duloxetine treatment are important given their potential contribution to efficiency.

Interestingly, overall effects were significant for the two executive functioning domains, but post hoc analyses did not yield significant differences on specific outcome measures. Examination of the individual difference scores suggests that for the set-shifting and working memory domain, changes were occurring in the expected direction, but there may have been insufficient power to detect differences at the level of individual outcomes. For the spatial planning domain, examination of the difference scores suggests a more complicated picture in that changes in planning time were inconsistent among problems with varying numbers of moves. This may be explained in part by the fact that measures of mean subsequent thinking time cannot be evaluated for persons who are slower in the yoked control portion of the task.

The results of this study are similar to those observed by Herrera-Guzmán et al. [35, 36] following duloxetine treatment in several ways but differ in some important ways as well. First, both studies noted improvements in psychomotor speed and mental processing that spanned several cognitive domains, with two of the individual tasks (RTI five-choice movement time and PRM mean correct latency) significantly improving in both studies and one (DMS mean correct latency) significantly improving in the current study and approaching significance (*P* = .051) in the Herrera-Guzmán study [35]. Herrera-Guzmán [35] found several significant improvements in visual memory, which were observed in a more limited manner in the current study (i.e., on the percent correct measure of one of the visual memory tasks). Both studies noted significant improvements in verbal learning as well, a finding that was also obtained by Raskin et al. [16] in their examination of duloxetine on cognitive function in elderly patients with MDD. The current study failed to see the expected improvements on individual tests of executive function that were observed in the Herrera-Guzmán et al. [36] study. However, we believe this is likely due to insufficient power in our sample given the fact that we found significant overall effects in the executive function domains as described above. Both studies also observed cognitive improvements that were independent of symptom severity. The similarities between these studies are noteworthy given the significant overlap in the design of the two studies including a similar age range and the use of the CANTAB, and they are also important in that the current study did not solely evaluate cognition in individuals who showed a clinical response to treatment.

Limitations of this study include the small sample size and open-label treatment. In addition, a nondepressed control group was not assessed for comparison of baseline performance. While participants were required to endorse cognitive difficulties for study eligibility, it is not necessarily true that this endorsement would reflect objectively measurable pretreatment difficulties in all of the cognitive domains assessed. Thus, absence of improvement in some domains may be the result of normal baseline performance to begin with, which cannot be addressed without a control group. As this was a first step in understanding how duloxetine may affect cognitive performance in individuals subjectively reporting cognitive difficulties, future studies are needed to examine the effects of duloxetine in patients with established objective cognitive impairment associated with their depression. However, in the current study, participants served as their own control and one could argue that it may be more important, or at least equally important, to determine improvements at the individual level rather than by comparison to healthy controls. There is a great deal of individual variation on task performance, and improvement is relative to how participants perform when they are not depressed. Thus, we believe that the meaningfulness of the results of this study lies in the fact that participants who before treatment endorsed impairment that was causing them difficulty in their daily lives showed improvement on cognitive tasks following duloxetine treatment, as well as subjective report that their cognition had improved. Defining improvement solely based on norms or comparisons to nondepressed controls may very well miss clinically meaningful improvements in cognitive function for depressed individuals. Another potential limitation of this study is the possibility that observed improvements were due to practice effects and/or the development of an efficient problem-solving strategy for the task (i.e., some tasks may have limited utility to detect changes once an effective problem-solving strategy is adopted and the task is no longer novel). Parallel forms were used for available tests in an attempt to avoid practice effects, but the possibility of practice effects cannot be completely eliminated, particularly for those tests for which only the clinical version was available.

As mentioned previously, an important aspect of this study is the use of a subjective (self-report) assessment to screen for cognitive impairment prior to treatment. Currently no gold standard screening approach exists for cognitive dysfunction that is representative of impairment on all domains thought to be associated with MDD. The approach used in this study follows what is most likely to be effectively implemented in clinical practice. This study evaluated both baseline subjective evaluation and clinician-rated evaluation of cognitive function, in addition to objective assessment of cognitive performance. Improvements were observed in all of these areas following treatment, suggesting that assessment of subjective functioning may be a useful proxy for assessing cognitive impairment. This is important given the practical implausibility of utilizing objective testing in a busy clinical practice. However, the results of this study are preliminary, and further work is needed to determine the best approach to measuring cognitive changes in response to treatment and the potential relationship between subjective and objective cognitive impairment in depression.

The results of this study support the use of duloxetine for individuals whose presentation of depression includes cognitive impairments. The lack of relationship between symptom severity and cognitive performance suggests that duloxetine may exert a positive effect on cognitive function independently of depressive symptoms, consistent with previous reports suggesting the same [36]. Therefore, it is conceivable that duloxetine may also be a potential augmenting agent for depressed individuals with residual cognitive symptoms following initial antidepressant treatment. Further examination of the potential benefit of duloxetine as a targeted treatment strategy for cognitive function in depression is recommended.

## Figures and Tables

**Table 1:** Description of cognitive function outcome measures.

Task	Acronym	Outcome measure	Description
Attention			
Big/little circle	BLC	Mean correct latency (ms)	Speed of response showing how quickly participant touched the correct stimulus after it was displayed on the screen
		Mean percent correct (%)	Percent of total correct responses
Reaction time	RTI	Five-choice movement time (ms)	Time taken to touch correct stimulus after release of the press pad
		Five-choice reaction time (ms)	Speed with which participant releases press pad in response to stimulus at one of five locations

Visual Memory			
Delayed matching to sample	DMS	Percent correct (all delays) (%)	Percent of correct stimulus selection after stimulus was hidden at delays of 0 s, 4 s, and 12 s
		Percent correct (simultaneous) (%)	Percent of correct stimulus selection during simultaneous presentation of target stimulus and distractors
		Mean correct latency (all delays)	Average speed of response where correct stimulus was selected in trials in which stimulus was hidden at delays of 0 s, 4 s, and 12 s
		Mean correct latency (simultaneous)	Average speed of response where correct stimulus was selected in trials with simultaneous presentation of target stimulus and distractors
Paired associates learning	PAL	Mean trials to success	Total number of trials required to correctly locate patterns/number of successfully completed stages
		Total errors (adjusted)	Total number of errors with adjustment for each stage not attempted due to previous failure
		Total trials (adjusted)	Total number of presentations required to correctly locate patterns in all stages
Pattern recognition memory	PRM	Percent correct	Percent correct responses
		Mean correct latency (ms)	Mean time to respond correctly

Executive Function/Set-Shifting and Working Memory			
Intra/extradimensional set-shifting	IED	Stages completed	Number of stages completed out of nine possible
		Pre-ED errors	Number of errors prior to the extradimensional shift
		EDS errors	Errors made in the extradimensional stage
Spatial working memory	SWM	Strategy	Number of times participant begins a search with the same box for 6- and 8-box problems
		Between errors (4, 6, and 8 boxes)	Times the participant revisits a box in which a token was previously found; errors calculated for 4-, 6-, and 8-box trials

Executive Function/Spatial Planning			
Stockings of Cambridge	SOC	Problems solved in minimum moves	Number of times participant successfully completed a test problem in the minimum possible number of moves
		Mean initial thinking time (2, 3, 4, and 5 moves)	Time taken to plan a problem solution for trials requiring 2, 3, 4, and 5 moves
		Mean subsequent thinking time (2, 3, 4, and 5 moves)	Speed of movement after the initial move has been made for trials requiring 2, 3, 4, and 5 moves

Decision Making and Response Control			
Affective go/no-go	AGN	Mean correct latency	Mean time taken to respond correctly to each target word stimulus in all assessed blocks.
		Total omissions	Total number of missed responses to targets in all assessed blocks
		Total commissions	Total number of responses to distractors in all assessed blocks

Verbal Learning and Memory			
Verbal recognition memory	VRM	Immediate free recall total correct	Total number of words correctly recalled immediately following presentation of word list
		Immediate free recall total novel words	Total number of words recalled immediately following presentation of word list that were not a part of the list
		Immediate free recall total perseverations	Total number of times a previously correctly recalled word is repeated immediately following presentation of word
		Immediate recognition total correct	Total number of words correctly recalled during presentation of word list that includes correct targets and distractors
		Immediate recognition total false positives	Total number of distractors endorsed as correct responses during presentation of word list that includes correct targets and distracters
		Delayed recognition total correct	Total number of words correctly recalled during presentation of word list that includes correct targets and distractors following 20 min delay from original presentation of word list
		Delayed recognition total false positives	Total number of distractors endorsed as correct responses during presentation of word list that includes correct targets and distractors following 20 min delay from original presentation of word list

**Table 2:** Participant baseline characteristics.

Baseline variable	Noncompleters (*N* = 9)		Completers (*N* = 21) (analyzable sample)	
	Mean or %	SD	Mean or %	SD
Age (years)	32.1	7.8	31.3	6.6
Male (%)	44.4		33.3	
Female (%)	55.6		66.7	
White (%)	66.7		57.1	
Black (%)	11.1		19.1	
Hispanic (%)	22.2		23.8	
Education (years)*	13.7	6.4	14.2	3.5
Characteristics of depression				
Age of onset (years)	22.3	8.0	18.8	7.6
Length of current episode (months)	58.9 (Median = 12.0)	132.8	36.5 (Median = 6.0)	60.0
Number of previous episodes	2.7	1.3	2.8^+^	2.0
Baseline symptom severity				
HRSD_17_	18.8		19.4	4.4
IDS-C_30_	35.8		36.8	8.7
IDS-SR_30_	36.2		37.9	12.1
Estimated intelligence				
NART-R	107.3		106.6	9.3

*n = 17.

^+^ *n* = 18; 3 participants not included because they indicated the number of previous episodes were too many to count.

SD: standard deviation; HRSD_17_: Hamilton Rating Scale for Depression, 17 items; IDS-C_30_: Inventory for Depressive Symptomatology–Clinician-Rated, 30 items; IDS-SR_30_: Inventory for Depressive Symptomatology–Self-Report, 30 items; NART-R: National Adult Reading Test-Revised.

**Table 3:** Performance on cognitive outcome measures before and after duloxetine treatment.

Task	Outcome measure	Pretreatment			Posttreatment			ES
		Mean (SD)	*z*-score mean	*z*-score (SD)	Mean (SD)	*z*-score mean	*z*-score (SD)	
BLC	Mean correct latency	726.1 (135.2)	0.07	1.12	684.7 (123.3)	0.38	1.06	
	Mean percent correct^a^	99.9 (0.6)	−0.19	1.51	100.0 (0.0)	0.16	0	

RTI	Five-choice movement time	**480.7 (108.3)**			**432.4 (91.5)**			**0.48**
	Five-choice reaction time	415.6 (121.4)			378.1 (52.3)			

DMS	Percent correct (all delays)	**83.2 (9.8)**			**94.3 (6.4)**			−**1.37**
	Percent correct (simultaneous)^a^	98.1 (6.0)			97.1 (7.2)			
	Mean correct latency (all delays)	**3656.2 (1058.4)**			**2958.8 (727.0)**			**0.78**
	Mean correct latency (simultaneous)	2926.9 (941.3)			2672.4 (573.3)			

PAL	Mean trials to success	1.6 (0.5)			1.6 (0.7)			
	Total errors (adjusted)	10.4 (10.0)			6.9 (8.0)			
	Total trials (adjusted)	8.0 (2.4)			7.8 (2.8)			

PRM	Percent correct	90.3 (13.3)			91.07 (11.9)			
	Mean correct latency	**1942.4 (514.8)**			**1730.4 (359.8)**			**0.48**

IED	Stages completed^a^	8.1 (1.9)			8.7 (0.6)			
	Pre-ED errors	9.6 (9.2)			7.5 (3.1)			
	EDS errors	6.4 (8.5)			7.3 (8.5)			

SWM	Strategy	34.8 (5.3)	−0.76	0.93	33.4 (5.5)	−0.52	0.95	
	Between errors 4 boxes	1.3 (2.4)	−0.43	1.45	0.8 (1.2)	−0.42	1.49	
	Between errors 6 boxes	6.9 (7.9)	−0.46	1.17	4.9 (5.2)	−0.09	0.82	
	Between errors 8 boxes	21.6 (13.5)	−0.48	1.04	17.2 (11.8)	−0.48	1.04	

SOC	Problems solved in minimum moves	8.5 (2.1)			9.2 (2.0)			
	Mean initial thinking time 2 moves	2445.6 (1732.3)	−0.28	1.46	2979.0 (2981.9)	−0.33	1.88	
	Mean initial thinking time 3 moves	6241.2 (4245.1)	−0.03	1.14	5181.1 (2600.0)	0.28	0.56	
	Mean initial thinking time 4 moves	6906.5 (3849.4)	0.46	0.75	8513.3 (6505.3)	0.26	1.17	
	Mean initial thinking time 5 moves	9283.5 (6218.4)	0.45	0.75	9072.6 (5555.2)	0.48	0.65	
	Mean subsequent thinking time 2 moves^a^	444.1 (314.2)	−0.24	0.34	370.7 (225.2)	−0.01	0.51	
	Mean subsequent thinking time 3 moves^a^	4826.6 (6091.8)	−6.99	7.02	716.4 (817.0)	−0.06	0.75	
	Mean subsequent thinking time 4 moves^a^	3185.8 (3656.9)	−0.74	1.63	1001.8 (866.8)	0.38	0.41	
	Mean subsequent thinking time 5 moves^a^	2932.8 (3270.5)	−0.82	1.53	1209.4 (1668.0)	−0.17	1.6	

AGN	Mean correct latency	557.3 (65.6)			543.0 (51.8)			
	Total omissions	**22.0 (14.0)**			**14.5 (9.1)**			**0.65**
	Total commissions^b^	24.3 (15.4)			19.5 (9.1)			

VRM^c^	Immediate free recall total correct	**6.2 (1.7)**			**7.2 (2.0)**			−**0.54**
	Immediate free recall total novel words^a,b,c^	0.3 (0.6)			0.0 (0.0)			
	Immediate free recall total perseverations^a,c^	0.2 (0.9)			0.1 (0.4)			
	Immediate recognition total correct^b^	22.7 (2.3)			23.7 (0.5)			
	Immediate recognition total false positives^a,c^	0.4 (1.1)			0.1 (0.2)			
	Delayed recognition total correct	22.3 (2.6)			23.0 (1.5)			
	Delayed recognition total false positives^a,c^	0.7 (1.0)			0.7 (1.1)			

Measures of time and latency are reported in milliseconds. Note that parallel forms were used when available. Normed scores (*z*-scores) are calculated for clinical mode tests (BLC, SOC, and SWM) and are presented when available.

%: percent change from pretreatment to posttreatment (note percent differences that were significant at *P* < .05 are bolded; *t* values and significance values reported within the text); SD: standard deviation; EDS: extradimensional shift; ES: effect size (Cohen's *d*); BLC: big circle/little circle; RTI: reaction time; DMS: delayed matching to sample; PAL: paired associates learning; PRM: pattern recognition memory; IED: intradimensional/extradimensional shift; SWM: spatial working memory; SOC: stockings of cambridge; AGN: affective go/no-go; VRM: verbal recognition memory.

^a^Not included in MANOVAs for their respective domains due to lack of variation in measures (BLC, DMS, IED, and VRM measures) or inability to obtain a measure for the item in the majority of cases (SOC mean subsequent thinking time measures). Note these SOC measures showed changes in the expected direction, indicating at least numerical improvement for those who had a nonzero value for the measure (i.e., their movement time was less than their thinking time).

^b^Approached significance (*P* = .06).

^c^Note that percent change was not calculated for the verbal recognition memory tasks of immediate free recall total novel words, immediate free recall total perseverations, immediate recognition total false positives, and delayed recognition total false positives due to the limited number of values other than 0.
